# Concept Analysis of Building Rapport in the ICU

**DOI:** 10.1111/nicc.70739

**Published:** 2026-10-23

**Authors:** Mia Eriksson, Alexandra Ullsten, Anna Nordin, Jan Nilsson

**Affiliations:** ^1^ Department of Health Sciences, Faculty of Health, Science, and Technology Karlstad University Karlstad Sweden; ^2^ The Intensive Care Unit Central Hospital Karlstad Sweden; ^3^ Center for Clinical Research and Education Region Värmland Karlstad Sweden; ^4^ School of Health Sciences Örebro University Örebro Sweden; ^5^ Section for Advanced Nursing, Faculty of Social and Health Sciences University of Inland Norway Elverum Norway

**Keywords:** building rapport, concept analysis, family members, intensive care

## Abstract

**Background:**

Building rapport, understood as establishing a meaningful and trusting relationship through interpersonal interactions between healthcare professionals and patients or their family members, was described in research as early as the 1960s. Although studies highlight its importance in clinical relationships, the concept remains largely undefined, with limited research on experiences of rapport among family members of intensive care unit patients. This concept analysis can help to provide conceptual clarity for future practice and research.

**Aim:**

This paper aimed to analyse and define the concept of ‘building rapport’ with ICU family members.

**Study Design:**

The concept analysis framework of Walker and Avant was used, following the systematic steps of identifying uses, attributes, antecedents, consequences and model cases. Data were obtained from Swedish and English dictionaries and peer‐reviewed articles identified through a systematic literature search and screening. Titles and abstracts were reviewed, and six studies were selected based on predefined inclusion and exclusion criteria.

**Results:**

This analysis clarifies the previously undefined concept of building rapport from the perspective of ICU family members. Building rapport is characterized by empathy and mutual understanding. Key antecedents include communication, time and relationships, while consequences are increased trust and the development of a care partnership. An empirical referent is a shift from family members as passive recipients to active participants in care.

**Conclusions:**

Key features of building rapport with ICU family members are empathy and mutual understanding, supported by communication, time and relationships. The findings suggest that rapport may facilitate family participation and care partnerships in intensive care. However, the findings should be interpreted in light of the limited empirical basis and the absence of formal quality appraisal.

**Relevance to Clinical Practice:**

Clarifying the concept of building rapport may support communication training, reflective practice and active listening strategies that strengthen partnerships with ICU family members.

## Impact Statements

- What is known about the topic
  ○ Building rapport is widely recognized as an essential component of healthcare practice, yet there is a need to further explore and understand rapport in the context of intensive care and from the perspective of family members.
- What this paper adds
  ○ This analysis is one of the first studies to explore the concept of building rapport with ICU families.
  ○ In this context, the concept is characterized by empathy and mutual understanding, while communication, time and relationships are central to creating a care partnership.
  ○ The findings indicate a shift towards greater involvement of families in care and highlight the increasing importance of the nurse–family relationship in the ICU context.

## 1 Background

The environment of the intensive care unit (ICU) is burdensome to both patients and their family members [1, 2, 3] and due to the critical condition of patients admitted to the ICU, family members encounter distressing scenes and unfamiliar equipment [4]. Further, the ICU environment is associated with a high risk of psychological problems, such as depression, anxiety and stress‐related disorders in family members [1, 2, 5, 6, 7, 8, 9]. This must be taken into consideration and acted upon by the patient's care team [6]. ICUs provide care to the most severely ill inpatients [10]. Intensive care, also called critical care [11], is complex and multifactorial [12]. It is an interprofessional specialty [11, 13], using various technologies to provide support for failing organ systems [11]. As part of clinical practice, healthcare professionals (HCPs) are expected to build rapport with both patients and their families [14, 15, 16, 17]. As early as 1963, Joyce Travelbee described rapport as a series of nurse–patient interactions that evolve into a meaningful nurse–patient relationship [18].

In ICUs in Sweden and other countries, family members are expected to be involved in patient care [13, 19], and HCPs are encouraged to develop collaborative relationships with families. This collaboration should be based on open information exchange, aiming to help families manage stress and advocate for the patient when necessary [20, 21]. Specialist ICU nurses lead the nursing care provided by the care team [13] and are responsible for ensuring a person‐centred approach to care [13, 22]. Supporting family members is considered a vital aspect of their responsibilities [23, 24, 25, 26, 27]; they are uniquely positioned to provide this support as they spend much time bedside with patients and family members [2, 27, 28, 29]. The complexity of their work also requires technical and scientific skills, which directly affect patients' life and death [30, 31]. Given the patient's critical condition, the nurse–family relationship in the ICU may replace the traditional nurse–patient relationship [28, 32]. However, high‐technology care environments, such as ICUs may pose challenges for building rapport in a person‐centred manner [33, 34, 35], and the reliance on technology has tended to sideline the human and emotional needs of patients, families and HCPs. This calls for renewed reflection on the necessity of rehumanizing medical care and strengthening relationships between patients, their family members and HCPs [36, 37].

In her early work, Travelbee [18] emphasized that what truly matters is how nurses spend their time with patients, and pointed out that many nurses require constructive advice in this area [18]. English [38] argues that HCPs can choose how to spend their time with patients, and that investing time in establishing rapport with patients and their families is effective and rewarding [3, 38, 39]. Various healthcare documents emphasize the importance of building or establishing rapport [40, 41, 42] and having a professional approach towards patients and their families [13]. Even the need for professional training in rapport building is highlighted [16, 43]. Studies emphasize the role of rapport in shaping particular outcomes, such as improved patient satisfaction, compliance and treatment outcomes, and decreased malpractice risk [44, 45]. However, up to one third of complaints concerning contact with clinicians deal with communication and rapport [46, 47, 48, 49] and such complaints are increasing [50].

Research is scarce on family members' experiences of building rapport. Studies have recently explored and attempted to define rapport from the perspective of patients and families receiving palliative care [14, 38]. Despite its recognized importance, the concept of building rapport remains largely undefined, particularly in the ICU context and from the perspective of family members, and HCPs may have their own opinion on what rapport means. This represents a gap in knowledge, as rapport is a central component of interactions between HCPs and family members in the ICU. However, when building rapport is defined and conceptualized consistently, it is easier to explore its impact on the experiences and outcomes of patients and their families [38]. Walker and Avant's framework was selected due to its structured and practice‐oriented approach to clarifying complex clinical concepts. Its use is well established in ICU‐related concept analyses, including studies on family engagement, family resilience and critical care phenomena, supporting its relevance in the present context [51, 52, 53]. Against this background, there is a need to conduct a concept analysis of ‘building rapport’ from the perspective of ICU family members, which can help to clarify the concept for HCPs and provide conceptual clarity for future practice and research.

## 2 Aim

The aim of this paper was to analyse and define the concept of ‘building rapport’ with ICU family members.

## 3 Design and Methods

### 3.1 Concept Analysis

The concept analysis framework of Walker and Avant [54] was used to examine the phenomenon of building rapport with family members in the ICU. This framework examines the structure and function of a concept using eight steps: (1) select a concept, (2) determine the aims or purposes of the analysis, (3) identify all possible uses of the concept, (4) determine the defining attributes, (5) identify model cases, (6) identify borderline, related, contrary, invented and illegitimate cases, (7) identify antecedents and consequences, and (8) define empirical referents. Although the procedure is presented in steps, the process is iterative, requiring substantial thought, reflection and revision of earlier steps because of information or ideas arising from a later step [54].

Concept analysis shares certain similarities with systematic reviews in that it seeks to identify as many uses of the concept as possible [54], whereas a systematic review aims to locate all records on a specific topic through an unbiased, transparent and reproducible search [55]. However, in contrast to a systematic review, which involves critical appraisal and evaluation of findings, or a scoping review, which seeks to identify and map the available evidence [56], concept analysis does not include a formal quality assessment of the sources when identifying the concept [54].

Concept analysis enables the researcher to consult dictionaries to identify the uses of a concept, and to move back and forth between languages in order to verify conceptual equivalence. This was particularly important in the present study, as the research question originated from a Swedish concept that does not readily lend itself to direct translation into English.

This procedure began by selecting a concept considered the equivalent of the Swedish concept ‘gott bemötande’, namely ‘good encounter’, which was analysed through Steps 1–5 above. However, this concept did not sufficiently capture the essence of what was to be explored. In Steps 3–5, research articles were analysed, and in one of these [57] the concept of building rapport was identified. This concept was also found to resonate more closely with the patterns observed in the data. Discussions with other researchers concluded that ‘building rapport’ was a more suitable match for the Swedish term. ‘Gott bemötande’ carries a distinctly positive connotation and may be difficult to equate with ‘encounter’, which may imply an element of conflict [58, 59]. Consequently, the procedure was repeated from the first step, but now with the concept of building rapport. Alternative conceptualizations were considered, but ‘building rapport’ was found to provide the most coherent fit, as it captures the relational and processual aspects of the phenomenon as described in the literature, where rapport is understood as a key element in establishing therapeutic or caring relationships [57, 60, 61].

The iterative nature of the concept analysis process helps to ensure a precise analysis. A concept analysis helps to clarify overused or vague concepts in nursing practice to enable all users of a term to have the similar understandings of its use [54].

### 3.2 Data Sources

In the early stages of the analysis, the main author and an academic librarian conducted a primary database search to identify concept analyses of ‘good encounter’. No previously published concept analysis was found. An initial scoping search was also performed to scan articles for the term and relevant surrogate terms. This scoping search was followed by a systematic literature search. Data sources included in the search were Swedish and English dictionaries and English language scholarly articles. This procedure was later repeated with the concept of ‘building rapport’; however, at the time this search also revealed no previously published concept analysis in English. Later on, a manual search resulted in one relevant article.

### 3.3 Dictionary Analysis

First, to identify all uses of the Swedish concept of ‘gott bemötande’, a Swedish dictionary analysis including an etymological and semantic part was performed in five Swedish dictionaries [62, 63, 64, 65, 66]. The Swedish concept was separated into two words and the origin and meaning were analysed. First, an etymological analysis of the concept's origin and history was conducted, followed by an analysis to identify its linguistic meaning. After this, a Swedish‐English dictionary [67] was used to find the English meaning of the two words (including synonyms). The English words were then checked in Swedish to ascertain that they had the desired meaning [68]. The terms were then searched for in three English dictionaries to find keywords, synonyms and related words. The synonyms or related words identified in different dictionaries were report [69, 70], account, write‐up, statement, message [69], understanding, empathy [70, 71, 72] and sensitivity [72].

### 3.4 Search Strategy and Databases

Included databases were CINAHL, Medline and PsychInfo. Search terms were text words and MeSH terms, depending on the databases (Supporting Information Appendix S1). The search had no time limit, because the analysis aimed to identify all uses of the concept (conducted on 30 January 2025).

### 3.5 Identification of Relevant Studies

Titles and abstracts were screened and articles included if the title or abstract contained various variations of the phenomenon of building rapport from the perspective of family members in the intensive care context. Studies were excluded based on the following criteria: focus on children, neonatal care, HCPs' perspective, patients' perspective, other contexts than intensive care and medical treatments. Articles that could not be retrieved in the English language were also excluded.

### 3.6 Selecting the Evidence

The database search led to 189 articles, as demonstrated in a PRISMA flowchart (Figure 1).

**FIGURE 1:**
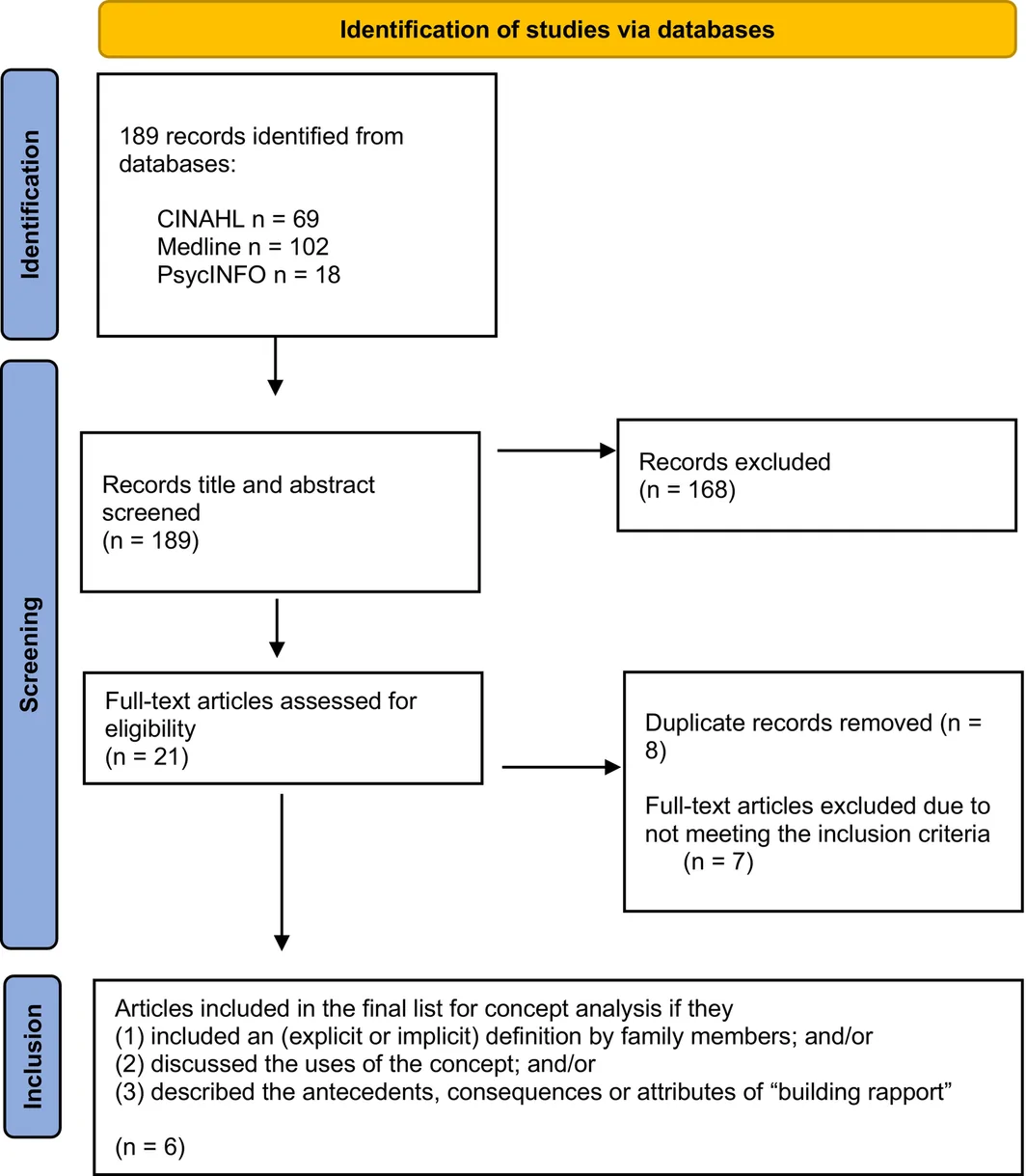
PRISMA flowchart of systematic literature search, showing the search for the concept of ‘building rapport’ (Based on Moher et al.) [73]. *Source*: Page MJ, et al. BMJ 2021;372:n71. doi:10.1136/bmj.n71. This work is licensed under CC BY 4.0. To view a copy of this licence, visit https://creativecommons.org/licenses/by/4.0/.

As the concept analysis framework [54] encourages identification of as many uses of the concept as possible, no critical appraisal of the included articles was conducted, as this could bias the understanding of the true nature of the concept.

### 3.7 Extracting the Evidence

Following the framework of Walker and Avant [54], the analysis initially consisted of creating an overview of the data and identifying the most common uses of the concept by organizing relevant textual content from the dictionaries and the included articles in tables. The dictionary content and empirical data were first analysed sequentially, starting with the dictionary sources, and subsequently iteratively by moving back and forth between the sources to compare similarities and differences in the uses and attributes of the concept. Thereafter, examples of the concept of building rapport were highlighted in the texts, including descriptions and definitions. Attributes were identified through careful attention to the characteristics and properties described in connection with building rapport in the different sources. These characteristics were compared across texts and grouped into categories based on similarities and differences. Two categories were considered as essential attributes of building rapport. To enhance analytical rigour, the identification and categorization of attributes were continuously discussed within the research team, allowing for critical reflection on interpretations and consensus regarding what constituted an attribute. The analysis also involved iterative movement between the original sources and the emerging categories to ensure that the findings were grounded in the data. Thereafter, a model case was identified. During the analysis of the articles, contrary, borderline and related cases were also highlighted to clearly distinguish the concept from other concepts. Necessary antecedents of the concept and consequences of the concept were identified in the data. Finally, empirical referents were defined.

## 4 Findings

### 4.1 Uses of the Concept of Building Rapport

The concept of building rapport as a phrase was not found in any of the dictionaries [74, 75, 76, 77, 78]. In the final set of included articles, building rapport as a phrase was found in three of six articles. The remaining three articles used variations, such as establishing rapport, family‐clinician rapport or greater rapport. The included articles were all qualitative studies.

Build, as a verb, means to construct or make [79], to create and develop over time, as in ‘build a relationship’ [80], and to form by ordering and uniting materials gradually into a composite whole [81]. As a noun, a building is something built or constructed [82]. The meaning of rapport in English is a good understanding of someone and an ability to communicate well with them [72]. Swedish translations of rapport are good conditions, good relations or good understanding [69]. Rapport involves mutual understanding between persons: sympathy, empathy and connection [70]. One definition of rapport is ‘a friendly, harmonious relationship, especially a relationship characterized by agreement, mutual understanding, or empathy that makes communication possible or easy’ [71].

In the early 20th century, English speakers borrowed ‘rapport’ from French in the meaning of a friendly, harmonious relationship. The following sentences exemplify possible uses of rapport: ‘She works hard to build rapport with her patients’ [71] or ‘Forming a good relationship and rapport with the patient and family is viewed as a critical pre‐requisite to quality care’ [72]. The dictionary meanings provide a semantic foundation that is reflected in the empirical findings, where rapport is described as a relational and communicative process characterized by mutual understanding and positive interaction. Although the dictionary definitions provide a linguistic understanding of the term, the empirical findings contribute a more theoretically informed interpretation of the concept within a healthcare context by demonstrating how rapport is understood, developed and experienced in interactions between HCPs and family members.

### 4.2 Building Rapport From the Perspective of ICU Family Members

Consistent with the dictionary definitions, the concept of building rapport in the intensive care context from the perspective of family members can be understood as a set of relational strategies that strengthen the therapeutic relationship, which includes holding family members in high esteem, being approachable and being affable [57]. Rapport can also be built over the phone [83]. The concept of rapport encompasses predominantly positive qualities, such as high ratings of communication and therapeutic alliance and low conflict [60]. In a relational aspect of care, rapport benefits patients, family members and the clinical team alike [84]. Trust and rapport in a care partnership are mentioned together [25, 85], and by family members. There are frequent references to the importance of building rapport [85]. These descriptions highlight rapport in a positive way and illuminate the relational aspect of care, which is in line with the Swedish concept of ‘gott bemötande’ and the purpose of this paper. These descriptions also emphasize that in the ICU context the traditional nurse–patient relationship tends to be replaced by a nurse–family relationship. Taken together, these findings indicate that building rapport in the ICU context involves a relational and communicative approach that supports trust, reduces conflict, and fosters a sense of partnership between family members and HCPs.

### 4.3 Defining Attributes

The core of a concept analysis is the defining attributes, that is, those most frequently associated with the concept, which differentiate the specific phenomenon from others [54]. In this analysis, the final attributes that define the concept of building rapport from the perspective of ICU family members are *empathy* [70, 71, 83, 85] and *mutual understanding* [70, 71]. The attribute empathy was reflected in both dictionary definitions [70, 71] and empirical descriptions of actively acknowledging families' emotional burden, recognizing their distress and understanding their challenges [83]. Although dictionaries emphasized mutual understanding [70, 71], the empirical findings expanded on this by showing that mutual understanding reflects a two‐way interaction: professionals strive to make complex information accessible, while actively listening to the patient and family and exploring what is meaningful to them [25, 85]. An additional dimension, not evident in the empirical data, emerged from lexical sources where building rapport is described as a close, harmonious relationship in which the people concerned understand each other's feelings and communicate well [72]. Although additional elements such as trust, communication and time are associated with rapport, these were identified as antecedents or consequences rather than defining attributes. The selection of empathy and mutual understanding as defining attributes was based on their presence in both lexical and empirical sources, as well as their central role in characterizing the core relational aspects of the concept from the perspective of ICU family members.

### 4.4 A Model Case

A model case may be an actual example from real life, a case found in literature or a constructed case. The model case is an example of the use of the concept and should demonstrate all the defining attributes of the concept to enable understanding of its core meaning [54].

Jenny's sister was critically injured in a car accident and was unconscious when admitted to the ICU. She was ventilated due to traumatic brain injuries. Jenny visited daily, and the nurses constantly reassured her that she could rest at home and that they were always accessible for updates. At the bedside, the nurses sat with Jenny and listened attentively as she recounted the accident. They comforted her when she cried and gently explained the signs indicating the seriousness of her sister's condition. To help her stay connected, they encouraged her to talk about her sister and share memories. Their conversations sometimes shifted to everyday topics, creating moments of normalcy amid uncertainty. Jenny was welcome to stay close, even during care routines. She watched the nurses reposition her sister with calm, respectful explanations, and they invited her to participate by combing her sister's hair and sharing details about her sister's preferences. These actions made Jenny feel understood, supported, and included in her sister's care.

### 4.5 Borderline Case

A borderline case has most but not all of the attributes, and an inconsistency, such as length of time or intensity of occurrence of the attribute [54].

Michael's partner's heart failure had worsened, and he was now in the ICU. During visiting hours, the nurses showed genuine empathy toward both of them, and even the doctor took time to answer Michael's questions. Michael felt seen, supported, and able to trust the team. But after visiting hours, everything changed. Having to leave made him deeply uneasy—what if his partner's condition worsened while he was gone? He wished he could stay just a little longer, at least until his partner fell asleep. The staff's compassion during visits contrasted sharply with the strict visiting rules, interrupting the sense of connection that had begun to form. Michael left each evening torn between trust in the caregivers and helplessness over having to leave.

### 4.6 A Related Case

A related case is connected to the concept but does not contain all the defining attributes. When examined more closely, this case demonstrates very similar ideas to those of the concept, but differs in a way that provides clarification of what counts and does not count as defining attributes of the concept [54].

Kristen's mother had lived with COPD for years but now required intensive care due to pneumonia. Everything happened quickly, and Kristen tried to understand what was going on. The nurses were efficient and focused on treatment, but they seemed rushed and took little time to talk. Although one nurse explained the care plan clearly, she avoided eye contact and her tone lacked warmth, leaving Kristen unsure if she could ask more questions. Polite as they were, the staff made Kristen feel like just another visitor. She stood by the bedside, hesitant to interrupt the busy routines. She trusted that her mother was receiving good care, yet she felt invisible and had no space to express how frightening the situation was for her. This was an encounter—functional and informative, but lacking the empathy and mutual understanding required to build rapport with family members.

### 4.7 A Contrary Case

A contrary case exemplifies what the concept is *not* [54].

John's wife had undergone major abdominal surgery and was now in intensive care. One day, John and his children met a nurse who appeared disengaged from the family. She did not introduce herself, stayed focused on the computer, avoided eye contact, and often turned her back to them. She offered no information, and when John asked a few questions, he felt that he was interrupting her. This left him uneasy and distrustful, unsure if his wife was in competent hands. He did not want to leave her bedside, yet felt helpless—watching someone he deeply cared for lying there vulnerable while being cared for by a nurse who showed no sign of empathy or understanding.

### 4.8 Antecedents

Events or incidents that must occur before the occurrence of the concept are antecedents [54]. Antecedents of building rapport are *communication*, *time* and *relationships*.

Good communication is key to building rapport [60, 85]. It involves honest and frequent communication [25], low conflict [60] and consistency [85]. Family members can receive affirmation by being encouraged to talk about the patient and themselves, ask questions and call. HCPs contribute through attentive listening, eye contact, sitting close, touching, small talk [57] and humour [57, 85]. In telephone communication, choice of words, tone, pitch, pacing and rhythm are essential [83]. Time is another central factor [25], including patience [57] and spending time with patients and families [85]. The relationships involved in building rapport resemble a therapeutic alliance [60], with family participation and a committed care team supporting the patient [84]. Such relationships build trust and recognize the values of families and patients [25]. Clinicians remain personable, demonstrating willingness to engage and admit mistakes [57]. Family members described needing to establish relationships with all the nurses [25] and viewed rapport as a form of contract with the care team [83].

### 4.9 Consequences

Consequences are the events or outcomes that occur as a result of the concept [54]. Outcomes of building rapport can be that family members feel personally connected with the nurse, trust the nurse and have more confidence in the nursing care [57]. It can also enhance their trust in the care team and reassure them that everything possible is being done and that nothing will be missed [84]. Nonverbal communication adapted to relatives' emotions was experienced as soothing [83], and humour helped ‘lighten the mood’ during an otherwise dark time [85]. Building rapport can also result in the sense of a care partnership [25].

### 4.10 Empirical Referents

By their presence, empirical referents demonstrate the occurrence of the concept [54]. A cultural shift from families as recipients of care to active participants in care [25] can be seen as an empirical referent of building rapport. In 1979, Molter [86] argued that the involvement of family members was essential to the care of ICU patients. By recognizing family members' important needs and assessing how they are being met, total patient care will involve the family [86]. To measure fulfilment of family members' needs and their satisfaction with the care provided to them and the patient, the Critical Care Family Needs Inventory [86, 87] and Family Satisfaction with Care in the ICU can be used [87, 88].

## 5 Discussion

This concept analysis contributes to the development of building rapport by examining the concept from the perspective of ICU family members, an area little studied. In this study, the attributes of building rapport are empathy and mutual understanding, which aligns with English et al. [14, 38], where mutual understanding is described as being accepted, being understood and understanding each other [14]. Empathy relates to the characteristics of caring, and family members need interactions with HCPs based on caring to develop rapport. Caring can be demonstrated through tone of voice, smiles, nods, knowing patients' names, interests and families, or sharing a laugh during interactions [14]. Empathy has previously been identified as a fundamental component of establishing rapport, in studies involving both HCPs and patients [15]. English et al. [38] also highlight definitions referring to relationships of empathy and mutuality [38]. Nadig [60] describes the relationships involved in building rapport with family members as therapeutic alliances, consistent with the broader literature on nurse–patient relations. Effective communication, honesty and respect, and the establishment of rapport and trust can lead to therapeutic relationships [61]. Nurses must keep in mind that providing assurance, comfort and support is an integral part of strengthening relationship‐centred care [89], which is consistent with recent ICU guidelines emphasizing the importance of structured communication and family‐centred care [90].

The antecedents, defining attributes and consequences identified in this analysis describe the phenomenon in context and provide a foundation for enhanced rapport building in intensive care. Although empathy and mutual understanding were identified as defining attributes in the present analysis, other studies have emphasized different elements, such as trust, communication and the therapeutic alliance, as important components of rapport [25, 60, 85]. This suggests that even within a similar clinical context, different aspects of rapport may be highlighted, indicating that the concept is multifaceted and open to multiple interpretations. Although terms such as empathy are difficult to quantify, English et al. [38] argue that defining and clarifying conceptualizations and operationalizations of rapport is essential for developing coherent knowledge and understanding. Establishing consistent conceptual and operational definitions enables HCPs to translate research findings into more effective clinical practices for fostering rapport [38]. Although existing literature describes the development of therapeutic relationships, there is less evidence on how nurses should use rapport in their profession [61]. Successful operationalization of building rapport in the ICU requires recognition of its antecedents, defining attributes and consequences. In practice, this may involve using structured approaches to communication, such as regularly scheduled family meetings and active listening techniques that explicitly acknowledge family members' emotional needs. In addition, existing instruments, such as the Critical Care Family Needs Inventory and measures of family satisfaction, may serve as proxies for evaluating aspects of rapport, although they do not directly capture the concept. Furthermore, established scales related to person‐centred care and intensive care nursing competence may provide a basis for identifying or adapting items relevant to the assessment of building rapport in clinical practice. Person‐centredness may support the formation of therapeutic relationships, but according to Andersson [34], specialist ICU nurses need knowledge of the philosophical underpinnings of person‐centredness before integrating it into care [34]. From a Ricoeurian standpoint, the ethical dimension of nursing care entails a fundamental desire for the good of the other, where mutuality is grounded in acknowledging the other as a unique person [91]. These philosophical perspectives can be seen as underpinning the defining attributes identified in this study, particularly empathy and mutual understanding, by highlighting the importance of recognizing the other as a person and engaging in meaningful relational communication.

Listening to families' stories about the patient can enable nurses to become familiar with the patient as a person [34, 92], while nurses' ability to be affected by the patient's suffering and respond emotionally is a prerequisite for mutuality [34]. In the literature, family members described a relationship comparable to a contract between the care team and themselves, which they found necessary to build rapport [25]. This aligns with principles of person‐centred care [92], where relational presence, attentiveness and responsiveness to patients and their families are central to care practices in the ICU context [93, 94]. Adopting a person‐centred approach, that is, taking the time to listen to the patient's narrative, establishing a partnership, achieving shared understanding and jointly formulating a care plan [95] may lead to benefits, such as reduced length of stay [96].

## 6 Limitations

Certain methodological limitations should be acknowledged. One concern is that the concept analysis framework of Walker and Avant [54] has been criticized for not explicitly articulating the philosophical or ontological assumptions [97, 98, 99]. However, contextualizing concepts strengthens their epistemological and ontological footing, as case descriptions can demonstrate contextual patterns of use and serve as the evidential basis for the concept [98]. Furthermore, if theory emerges from attempts to solve practical problems, conceptual work gains legitimacy when situated within practice contexts, often captured through context‐specific cases [99]. The present study does not aim to develop theory but to provide a systematic and transparent initial survey of usage; here, Walker and Avant's [54] procedure offers a clear, reproducible foundation for later theoretical elaboration.

The review of the literature supports and validates the choices of the defining attributes, while also providing the evidence base for the analysis [54]. The included databases were CINAHL, Medline and PsycINFO; focusing on subject‐specific databases enabled the use of controlled vocabulary and improved precision. An experienced librarian ensured comprehensive coverage of the search terms. Even though the limited use of ‘nurse’ as a search term may be considered a weakness, the contextual search strategy successfully identified studies involving other ICU professionals, such as physiotherapists [85].

Although the analysis was based on six scientific articles, these were strategically selected for their conceptual relevance. In contrast to empirical studies aiming at statistical generalization, concept analysis prioritizes depth of understanding and theoretical insight. The material was considered sufficient because recurring patterns and attributes of the concept could be identified, indicating conceptual adequacy. Thus, the limited number of sources should not be interpreted as a weakness but seen as consistent with the methodological approach. Despite the identification of recurring patterns, the limited number of studies may have restricted the attainment of full conceptual saturation, suggesting that additional attributes or variations of the concept may not have been captured.

These limitations may have influenced the interpretation of the findings, as the limited number of studies and their cultural context could have shaped which aspects of rapport were emphasized, whereas others may have remained underrepresented. The limitations may also have influenced the findings by shaping how the defining attributes were interpreted and delimited, particularly given the reliance on a limited number of sources and the interpretive nature of the analysis. In addition, the absence of critical appraisal might have allowed studies of varying quality to contribute equally to the conceptualization.

Finally, the previous review of rapport regarding patients, families and HCPs [38] was primarily based on studies from Western countries, which may limit the ability to identify cultural variations in how rapport is understood or enacted. In the present study, the exclusion of non‐English studies and the reliance on Swedish and English sources may have introduced cultural bias and limited the transferability of the findings. This may influence how rapport is understood and manifested in different cultural contexts, as relational norms and communication practices can vary, potentially limiting the applicability of the findings beyond similar settings.

The use of model (fictional) cases, while enhancing clarity, also entails certain limitations that may influence the study's credibility. As Risjord [98] emphasizes, concepts derive their meaning from context and actual usage. Constructed cases are therefore not empirically grounded in the same way as real‐world observations and may reflect an idealized or normative representation of the concept. In the present study, this implies that the model cases of rapport could have simplified its complexity and not fully captured its variability in clinical practice. However, the included articles also provided empirical examples of inadequate rapport [57, 83] and additional studies illustrated various forms of impaired rapport [14, 100, 101, 102]. Consequently, while useful for illustration, model cases should be interpreted with caution in relation to empirical grounding [98]. This may have limited the extent to which the constructed cases reflected the full complexity and variability of rapport in clinical practice and could thus have influenced the credibility and transferability of the findings.

## 7 Implications for Practice and Further Research

To further develop an operational definition of the concept of building rapport, a research instrument that accurately reflects the defining attributes of the concept should be identified [54]. However, as English [38] identified the need for further research capturing families' experiences of rapport, it would be relevant to continue exploring ICU families' perceptions of rapport. Such research may help to validate and refine the defining attributes identified in the present concept analysis by exploring how rapport is experienced and understood by family members in intensive care settings.

In addition, further research is needed to examine how building rapport is reflected in intensive care nursing practice, including the development and testing of instruments to assess competence in relation to its defining attributes. This is important because although the present study provides a conceptual understanding of rapport, further empirical research is needed to explore how its defining attributes are manifested and recognized in clinical practice. Such work may lead to the implementation of strategies to strengthen rapport in clinical practice, for example, by supporting education, reflective practice and the development of communication strategies in interactions with family members. Future research should also examine the outcomes of rapport in intensive care, including its potential influence on trust, family participation, care partnerships and experiences of care. Such knowledge may help inform educational initiatives and evidence‐based strategies aimed at strengthening relationships between nurses and family members.

## 8 Conclusion

The concept of building rapport with family members in the ICU context is characterized by empathy and mutual understanding, where communication, time and relationships are central to creating a care partnership. Although these attributes define the core of the concept, rapport also appears to be a multifaceted phenomenon shaped by relational and communicative processes within the ICU context. Building rapport may facilitate person‐centred care and greater family involvement in care. Future research should continue to explore how building rapport can be understood, assessed and applied in clinical practice; however, the findings of this study should be interpreted in light of the limited empirical basis of the analysis.

## Author Contributions

Study conception/design, critical revisions for important intellectual content and drafting of manuscript: Mia Eriksson, Alexandra Ullsten, Anna Nordin and Jan Nilsson. Data collection and data analysis: Mia Eriksson.

## Funding

This work was supported by the Region Värmland, Sweden.

## Ethics Statement

The authors have nothing to report.

## Consent

The authors have nothing to report.

## Conflicts of Interest

The authors declare no conflicts of interest.

## Supporting information


**Appendix S1:** Searches in Medline 2025‐01‐30.

**Data S1:** Supporting Information.

## Data Availability

Not applicable for this concept analysis.
