# Supplementary material for: Concept Analysis of Building Rapport in the ICU
Source: Nurs Crit Care. 2026 Oct 23;31(6):e70739. doi: 10.1111/nicc.70739 (PMC13692800; doi:10.1111/nicc.70739)
Supplement: Supplementary file 1 [file NICC-31-0-s001.docx]

**PRE-SUBMISSION CHECKLIST FOR AUTHORS**

*Dear Authors,*

*Please confirm all the following points, indicate in which page number they are addressed and submit this form with your manuscript.*

*Many thanks,*

*The Editors*

|  | **Indicate Yes / No / NA (not applicable) and the manuscript page number in which this item is addressed** | **Comments (in case of NO or NA)** |
| --- | --- | --- |
| **Authorship & Anonymisation** |  |  |
|  |  |  |
| I have reviewed the [Wiley Generative AI policy](https://www.wiley.com/en-us/publish/article/ai-guidelines/) and, if relevant, I have included a “Declaration of generative AI and AI-assisted technologies in the manuscript preparation process” in the Methods or Acknowledgements section. This should include, but not be limited to reporting the model, version, and manner in which AI was used, and a statement that all authors reviewed the final manuscript and are responsible for the manuscript | Yes |  |
| The names of the authors, participants and the facilities in which the study was conducted do not appear anywhere in the manuscript or in the response to the reviewers. | Yes |  |
| The country in which the study was conducted is indicated in the methods section of the abstract and of the main text. | No |  |
|  |  |  |
|  |  |  |
| **Title Page** |  |  |
|  |  |  |
| A title page is uploaded separately | **Yes** |  |
| The title page matches the information provided in the submission system and includes:   - Manuscript title - Full author names, affiliations, accurate and active email addresses - Clearly identified corresponding author (including ORCID ID) - Funding statement - Conflict of interest statement - Ethics approval details (committee name, approval number, & date) - Patient consent statement - Data availability statement | **Yes** |  |
|  |  |  |
| **Abstract and Impact Statements** |  |  |
|  |  |  |
| *For full-length manuscripts (except case studies):* The abstract is structured, does not exceed 300 words and includes the following subheadings: Background; Aim (or Aims); Study Design; Results or Findings; Conclusions; Relevance to Clinical Practice. | *Yes* |  |
| *For case studies and short manuscripts*: The abstract is unstructured and does not exceed 200 words. | *NA* |  |
| The manuscript includes a set of impact statements, in the form of bullet points under the headings *What Is Known About This Topic* and *What This Paper Adds*. | Yes |  |
|  |  |  |
| **Language, Word Count and Abbreviations** |  |  |
|  |  |  |
| The manuscript is written in UK English spelling | No | American english |
| Abbreviations are written in full the first time in the Abstract, Main Text and Impact statement, followed by the abbreviation in brackets | Yes |  |
| The word count in the abstract and main text does not exceed the word limit **for my manuscript category**, indicated in the [author guidelines](https://onlinelibrary.wiley.com/page/journal/14785153/homepage/forauthors.html). | No | Word count may exceed after revision |
| The correct manuscript category is selected. | Yes |  |
| **Section Headings and Content** |  |  |
|  |  |  |
| The abstract and main text include a section called *Aim* or *Aims* depending on whether one or more aims are included. | Yes |  |
| *For quantitative studies*: “Results” (not “Findings”) is used as a section heading in the abstract and main text. | *NA* |  |
| *For qualitative studies*: *Findings* (not *Results*) is used as a section heading in the abstract and main text. | *Yes* |  |
| The *Discussion* section indicates where (in which country) the cited studies were conducted. | NA |  |
| The *Conclusion* is the last section of the manuscript. | Yes |  |
| The manuscript is structured according to the general [author guidelines](https://onlinelibrary.wiley.com/page/journal/14785153/homepage/forauthors.html) and those specific to my manuscript category. | Yes |  |
|  |  |  |
| **Ethical Approval and Registration** |  |  |
|  |  |  |
| Ethical approval was obtained before participant recruitment in an intervention study | NA |  |
| Ethical approval (if applicable) is reported anonymously at the end of methods section. The date of ethical approval is indicated in the DD MM YYYY format. | NA |  |
| *For clinical trials in which patients are randomised*: a **prospective** trial registration number is provided. This registration number is **not** anonymised. | *NA* |  |
| *For systematic reviews:* the registration number (if applicable) is **not** anonymised | *NA* |  |
|  |  |  |
| **Reporting Results** |  |  |
| *For research articles*: I have reported the dates between which the study was conducted. | NA |  |
| *In the case of studies reporting inferential statistics*: the data analysis section indicates how data were assessed for normality. | NA |  |
| *For quantitative studies*: the number of participants ***is reported in the Results section (not in the Methods section)*** both in the abstract and the main text. | *NA* |  |
| *For quantitative studies*: I have followed the journal’s guidance on reporting statistical analyses, confidence intervals, and statistical significance, including but not limited to the following:   - Confidence intervals are presented and may be used as the primary basis for inference without reliance on p-values. - Where p-values are reported, the corresponding measure of effect or association (e.g., Odds Ratio, Weighted Mean Difference, Cohen’s d, correlation coefficient, η²/ηp²) and its confidence interval are reported for each test, including in the abstract. - Exact p-values are reported to three decimal places, not merely as p <0.05. - Numbers are consistently reported as absolute and relative e.g. “n=5 (15%)” | *NA* |  |
|  |  |  |
| **Tables and Figures** |  |  |
| Tables do not exceed one page. Tables longer than one page are presented as supplementary material. | No |  |
| Any abbreviations used in a table or figure are defined at the bottom of the table / figure. | Yes |  |
| For all manuscript types, including bibliometric analysis, figures should include properly capitalised author and country names and official names of countries. | Yes |  |
| Tables, figures, and supplementary files should be uploaded in their designated sections. | Yes |  |
| If a supplementary file is intended for publication, it is cited within the manuscript. If it is not meant for publication, it is uploaded as “Not for Review or Publication” and is not cited in the text. | Yes |  |
|  |  |  |

**Acknowledgements**: This checklist is partly adapted from pre-submission checklist of the International Journal of Nursing Studies (IJNS). Thanks to Professor Peter Griffiths, IJNS Editor in Chief, for his permission.
