# Supplementary material for: Concept Analysis of Building Rapport in the ICU
Source: Nurs Crit Care. 2026 Oct 23;31(6):e70739. doi: 10.1111/nicc.70739 (PMC13692800; doi:10.1111/nicc.70739)
Supplement: Supplementary file 2 [file NICC-31-0-s002.docx]

**Supplementery Appendix S1. Searches in Medline 2025-01-30**

| **Concept/block 1** | | |
| --- | --- | --- |
| **Search number** | **Search terms** | **Search results** |
| S1 | "Critical Care"[Mesh] OR "Intensive Care Units"[Mesh] OR "Critical Care Nursing"[Mesh] OR "Critical Illness"[Mesh] OR "critical care" OR "critical care nursing" OR "Critically ill" OR "intensive care unit" OR ICU OR “intensive care” Title/Abstract | **570 835** |
| **Concept/block 2** | | |
| **Search number** | **Search terms** | **Search results** |
| S2 | "next of kin" OR spouse OR family OR "close relative" OR "patient family" OR "extended family" OR "nuclear family" OR daughter OR son OR sibling OR parent OR "adult child" OR relative OR "visitor to patient" OR relative OR husband OR wife OR family member All fields | **3 144 186** |
| **Concept/block 3** | | |
| **Search number** | **Search terms** | **Search results** |
| S3 | Rapport | **13 154** |
| **Combination of blocks** | | |
| **Search number** | **Search terms** | **Search results** |
| S4 | S1 AND S2 AND S3 | **102** |
